# Newly Generated Ca-Feldspar during Sintering Processes Enhances the Mechanical Strength of Coal Gangue-Based Insulation Bricks

**DOI:** 10.3390/ma16227193

**Published:** 2023-11-16

**Authors:** Yangfan Zheng, Jiayan Cui, Pengxiao Gao, Junfan Lv, Lin Chi, Hongyan Nan, Yuandong Huang, Fan Yang

**Affiliations:** 1 School of Environment and Architecture, University of Shanghai for Science and Technology, Shanghai 200093, China; 2 School of Chemical Engineering, Zhengzhou University, Zhengzhou 450001, China

**Keywords:** solid waste, coal gangue, porous structure, thermal conductivity, aluminosilicates

## Abstract

Coal gangue is a solid waste with low carbon content discharged during the course of the coal mining process. The resource utilization of coal gangue could solve environmental problems caused by its excessive production, such as soil contamination and land occupation. This study proposed to produce high-strength thermal insulation bricks using coal gangue as the primary material and three other mineral powders as auxiliary materials, including K-feldspar, CaCO_3_ and fly ash. A systematic analysis was conducted to explore the optimum raw material addition ratio and optimum sintering temperature; then, the intrinsic structure of thermal insulation bricks and their sintering formation mechanisms were revealed. The results showed that the optimal ratios of coal gangue, K-feldspar, CaCO_3_ and fly ash were 65 wt%, 15 wt%, 10 wt% and 10 wt%, respectively; the compressive strength of the thermal insulation brick produced under this ratio was 22.5 MPa; thermal conductivity was 0.39 W m^−1^ k^−1^. During sintering processes, mineral powders sufficiently fused to form a skeleton, and the CO_2_ derived from CaCO_3_ formed pores. The optimum sintering temperature was 1150 °C, because at this temperature, K-feldspar had the best effect in promoting the conversion of CaCO_3_ to Ca-feldspar. The high level of the relative crystallinity of Ca-feldspar (about 76.0%) helped raise the Si–O network’s polymerization degree (NBO/T = 1.24), finally raising the compressive strength of thermal insulation bricks. The innovative method of using coal gangue to make thermal insulation bricks not only solved the environmental pollution caused by coal gangue but also provided excellent construction materials with high practical application value.

## 1. Introduction

Coal gangue is an industrial waste from coal mining [1]. In China, coal gangue is one of the largest industrial solid waste types in terms of annual emissions and accumulation [2]. At present, the annual output of coal gangue in China is about 300~350 million tonnes, and the accumulated coal gangue is about 6~7 billion tonnes [2,3]. The substantial accumulation of coal gangue not only occupies farmland but also causes serious pollution to soil and groundwater. For instance, harmful soluble salts and toxic heavy metals in piled-up coal gangue seeped into the soil with rainwater, causing soil salinization and crop yield reduction and endangering human health via bio-accumulation [4]. In recent years, people conducted extensive research on the resource utilization of coal gangue, including using gangue as fuel [3], extracting chemical raw materials [5], and making building materials [6,7]. Among them, using gangue to make bricks is a promising research area [8]. However, bricks produced using only coal gangue often have disadvantages, including low compressive strength and poor thermal insulation. Process optimization is necessary to produce coal-gangue-based bricks with high strength and good thermal insulation.

Mixing coal gangue with other raw materials according to a certain proportion is feasible. They are then sintered together in thermal insulation bricks. Previous studies showed that the addition of fly ash helped improve the mechanical properties of sintered bricks [9]. Xu et al. [10] mixed fly ash with coal gangue to produce thermal insulation brick, and they found that the compressive strength, acid–alkaline resistance, and freeze–thaw resistance were all improved. The formation of a porous structure within the brick usually improved thermal insulation performance, and parameters such as porosity, cumulative pore volume and average pore size played an important role in thermal insulation performance [11]. CaCO_3_ is an expansion agent. Under high-temperature conditions, CaCO_3_ is decomposed and produces CO_2_ gas, which promotes the development of pores inside the bricks. Martínez-Martinez [12] studied the effect of sintering temperatures on the properties of clay ceramics prepared from sediments, and they found that CaCO_3_ was decomposed by heating and produced CO_2_, promoting the formation of porous structure. However, internal pores inevitably led to a decrease in brick density and mechanical strength [13], which could be possibly solved by fluxes such as K-feldspar. Fluxes not only reduced the sintering temperature of bricks but also reacted with other raw materials to improve the mechanical properties of bricks. Mao et al. [14] added K-feldspar fluxes to electroplating slurries to make bricks, and they observed that when the addition ratio of flux was 30 wt%, the compressive strength of bricks was doubled, and water absorption was reduced by about 50%. Therefore, with coal gangue as the primary raw material and fly ash, CaCO_3_ and K-feldspar as auxiliary materials, it was possible to produce high-strength thermal insulation bricks under optimal proportioning conditions.

The sintering temperature was another important factor that affected the mechanical strength and thermal insulation properties of bricks. Cheng et al. [15] prepared pavement bricks using Yellow River silt, coal gangue, and air-floating algal slag, and they found that the compressive strength of the bricks increased gradually with respect to sintering temperatures, reaching 20.8 MPa after 1100 °C. Mandal et al. [16] prepared insulated bricks by mixing fly ash and wood chips and suggested that the thermal conductivity of insulated bricks increased from 0.43 W m^−1^ k^−1^ to 0.63 W m^−1^ k^−1^ when sintering temperatures increased from 1000 °C to 1200 °C. The potential mechanism was that sintering temperatures changed the densities, phase compositions and micro-structures of bricks [6]. An increase in sintering temperature promoted the melting of mineral powders and improved the densification of bricks, Eliche-Quesada et al. [17] prepared lightweight bricks by mixing wood chips, marble and clay, and they observed that when sintering temperatures increased from 950 °C to 1050 °C, mineral powders reacted sufficiently to produce more liquid-phase compositions, promoting densification and an increase in compressive strength from 38.3 MPa to 84 MPa. For the effect of sintering temperatures on pore structure, Cultrone et al. [18] investigated the effect of sawdust on the physical properties of solid bricks and found that as sintering temperatures increased from 800 °C to 1100 °C, more silicate components were generated, which increased the vitrification degree of bricks, finally resulting in the destruction of the bricks’ compact pore structure. 

The aim of this study was to produce high-strength thermal insulation bricks via the sintering method, using coal gangue as the primary material and a range of mineral powders as auxiliary materials. A series of experiments were conducted: (1) three mineral powders, K-feldspar, CaCO_3_ and fly ash, were selected as auxiliary materials and mixed with coal gangue powder to produce high-strength thermal insulation bricks; (2) the raw material mixing ratio and sintering temperature were optimized based on a systematic evaluation, including compressive strength, flexural strength, water absorption rate and thermal conductivity; (3) the formation mechanisms of the Si–O network and the porous structure in insulation bricks were revealed via various chemical and spectral analyses.

## 2. Materials and Methods

### 2.1. Production of Coal-Gangue-Based Thermal Insulation Bricks 

Coal gangue was collected from Hebei Province, China. Fly ash was collected from thermal power plants. CaCO_3_ and K-feldspar were purchased from Sinopharm Chemical Reagent Co., Ltd. (Shanghai, China). The above materials were all passed through a 200-mesh sieve. Coal gangue powder was mixed with K-feldspar, CaCO_3_ and fly ash in a certain ratio, deionized water was added at a liquid–solid ratio of 7:10 (wt/wt), and they were stirred at 1000 rpm for 3 min until a slurry was formed. the slurry was transferred into a 40 × 40 × 40 mm mold. The mold was placed into an electric thermostat incubator at 60 °C for 48 h; then, it was demolded to acquire brick embryos. The brick embryo was placed into a muffle furnace and heated from 25 °C to 1150 °C at a rate of 5 °C min^−1^, and it stayed at 1150 °C for 1 h. After cooling to room temperature, the brick was taken from the muffle furnace.

As for three auxiliary materials, fly ash was thought to increase brick strength, so it was present in each group of treatments, and its addition ratio was maintained at 10 wt%. The addition ratio of K-feldspar and CaCO_3_ could significantly affect the internal structure and formation mechanism of bricks due to their fluxing and perforating effects. Therefore, this study proposes the exploration of an optimal formulation by varying the ratio of K-feldspar and CaCO_3_. Firstly, the ratio of CaCO_3_ is fixed at 20 wt%, and the ratio of K-feldspar was changed from 0% to 20 wt% (Table 1). After the optimal addition ratio of K-feldspar was identified at 15%, the ratio of K-feldspar was fixed at 15 wt%, and the ratio of CaCO_3_ was changed from 0% to 20 wt% (Table 1). In the experiment, to explore optimal sintering temperatures, the sintering temperatures were set to 1000 °C, 1050 °C, 1100 °C, 1150 °C and 1200 °C. The names of treatments were abbreviated: for example, K00-Ca20 meant that the addition ratios of K-feldspar and CaCO_3_ were 0% and 20 wt%, respectively, and T-1000 meant that the sintering temperature was 1000 °C.

### 2.2. Performance Testing of Coal-Gangue-Based Thermal Insulation Bricks 

In the compressive strength test, the insulated brick was placed perpendicular to the pressurized surface at the center of the press machine (JIANYI TYE-20B), and pressure was loaded smoothly and uniformly at a rate of 5 kN s^−1^ until the brick was crushed. The maximum fracture load was recorded, and compressive strength was calculated according to the following formula [19]:

$$R_{P}=\frac{P}{L\times B}$$

where R_p_ denotes the compressive strength (MPa), P denotes the maximum fracture load (N), and L and B denote the length and width of the compressed surface, respectively (mm). 

In the flexural strength test, a 40 × 40 × 160 mm mold was used to produce long thermal insulation bricks. Long thermal insulation bricks were laid flat on support rollers and uniformly loaded at a speed of 20 N s^−1^ until the thermal insulation bricks were fractured. The maximum fracture load was recorded, and flexural strength was calculated according to the following formula [12]:

$$R_{c}=\frac{3PL}{2BH^{2}}$$

where R_c_ denotes the flexural strength (MPa), P denotes the maximum fracture load (N), L denotes the distance between two support rollers (mm), and B and H denote the width and height of the brick, respectively (mm). 

The thermal insulation brick was placed in an oven, and drying took place at 105± 5 °C until a constant weight (m) was achieved. The size of the brick was measured to calculate volume (v). Bulk density (ρ) was calculated according to the following formula [20]:

$$\rho =\frac{m}{v}\times 10^{9}$$

where ρ denotes the bulk density (kg m^−3^); m denotes the dry mass of the thermal insulation block (kg); and v denotes the volume of thermal insulation block (mm^3^)

The thermal conductivity of thermal insulation bricks was measured via the transient flat plate heat source method using a thermal conductivity instrument (TPS2500S, Hot disk, Göteborg, Sweden), and the test temperature was set at 25 °C. Linear shrinkage was calculated by measuring the difference in brick size before and after sintering using vernier calipers. As for the water absorption test, the thermal insulation brick was placed in a 5 L beaker, and deionized water was added into the beaker until the water surface was 5 cm higher than the top of the brick. After immersing for 48 h, the thermal insulation brick was taken out and immediately weighed and then dried in an oven at 105 ± 5 °C until constant weight. The water absorption ability was calculated using the difference of two values. 

### 2.3. Characterization of Pore Structure and the Solid Phase Composition of Thermal Insulation Bricks

The chemical components of coal gangue and three auxiliary materials were analyzed via X-ray fluorescence (XRF-1800, Shimadzu, Kyoto, Japan). Before FTIR and XRD analyses, the insulation brick was ground into powder. The mineral compositions of insulation brick were detected via XRD, and the device utilized Cu K*α* radiation; the detection was conducted at 40 kV and 30 mA (D/max-2200/PC, Rigaku, Akishima, Japan), and the data were collected with 2*θ* ranging from 10° to 70° at a scan speed of 2° min^−1^. The functional groups of insulation bricks were detected via FTIR spectra (IR Prestige 21 FTIR, Shimadzu, Kyoto, Japan) using KBr pellets after 32 scans at a resolution of 4.0 cm^−1^. Four peaks of the FTIR curve were fitted to calculate the ratio of non-bridging oxygen to total oxygen (NBO/T) [21]. The pore structure of thermal insulation bricks was tested using an automatic Hg-pressure instrument (Mike Autoporelv 9510, USA) with a pore size range of 5~340,000 nm. A scanning electron microscope with an energy-dispersive spectrometer (Zeiss Gemini 300, Oberkochen, Germany) was used for micro-structural analysis.

### 2.4. Statistical Analysis

All treatments were carried out in triplicate, and the data were analyzed using Origin Pro 8.5.1, which had the following functions: data fitting, peak splitting, and peak area calculation. Suspicious values were processed according to the Q-value test. All significant differences were at a confidence level of 0.05, and all experimental data were presented as average ± standard deviations.

## 3. Result and Discussions

### 3.1. K-Feldspar Promotes the Conversion of CaCO_3_ to Ca-Feldspar

When the effect of K-feldspar/CaCO_3_ addition ratios on the performance of thermal insulation bricks was investigated, all bricks were prepared at 1150 °C. The main constituents of coal gangue, K-feldspar and fly ash were SiO_2_ and Al_2_O_3_. K-feldspar had the highest SiO_2_ content of 64.1% and the lowest Al_2_O_3_ content of 17.6% (Table 2), and its molecular formula was K_2_O·Al_2_O_3_·6SiO_2_. The Si–O tetrahedra and Al–O octahedra could form lattice structures comprising aluminosilicates in the thermal insulation bricks [22]. In addition, the content of K_2_O in K-feldspar was the highest (8.86%), which could play a fluxing role during the sintering process [23]. Among these three materials, coal gangue and fly ash had higher contents of Fe_2_O_3_ (6.16% and 8.80%, respectively) than K-feldspar (2.64%), because the former two are by-products of coal mining and coal combustion industries, respectively, and there was a large amount of Fe in coal [19]. 

**Figure 1:**
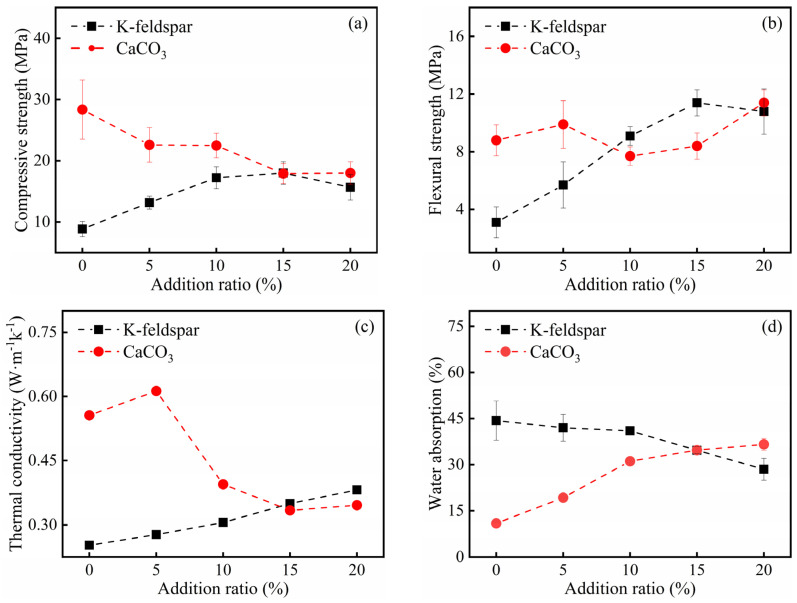
The compressive strength (**a**), flexural strength (**b**), thermal conductivity (**c**) and water absorption (**d**) of insulation bricks produced at different K-feldspar and CaCO_3_ addition ratios (K-feldspar: fixed CaCO_3_ addition ratio of 20 wt%; CaCO_3_: fixed K-feldspar addition ratio of 15 wt%).

The performances of thermal insulation bricks produced under different ratios of K-feldspar and CaCO_3_ were tested (Figure 1). When the ratio of CaCO_3_ was fixed at 20 wt% and the ratio of K-feldspar increased from 0% to 15 wt%, the compressive strength and flexural strength of insulating bricks increased from 8.87 MPa to 18.5 MPa and from 3.1 MPa to 11.4 MPa, respectively. However, when the ratio of K-feldspar continued to increase to 20 wt%, the compressive and flexural strengths slightly decreased. The above results indicated that when the ratio of K-feldspar was fixed at 15 wt%, the mechanical strength of thermal insulation bricks was the highest. With the increase in K-feldspar, thermal conductivity increased from 0.25 W m^−1^ k^−1^ to 0.38 W m^−1^ k^−1^, while the water absorption decreased from 45.0% to 28.0%, and this is probably due to the melting of K-feldspar powder, which filled the pores of thermal insulation bricks during the sintering process [24]. With an increase in CaCO_3_ from 0% to 20 wt%, compressive strength decreased from 28.4 MPa to 18.0 MPa, and flexural strength almost had no significant correlation (Figure 1a,b). The K15-Ca05 and K15-Ca20 treatments showed greater flexural strength, which may be related to the porosity and production of crystalline materials. Adediran et al. [25] prepared clay bricks by incorporating waste glass and coconut shells into clay and found that the incorporation of waste glass reduced the spacing between the particles, the mullite generated promoted the bonding of particles, and flexural strength was found to be 1.42 MPa. The water absorption increased from 11.0% to 33.0% (Figure 1d), and thermal conductivity firstly increased to 0.61 W m^−1^ k^−1^ (K15-Ca05) and then decreased to 0.33 W m^−1^ k^−1^ (K15-Ca15) (Figure 1c). This is because, at high temperatures, CaCO_3_ decomposed and released CO_2_, which promoted the formation of porous structure. The above result is similar to a previous study carried out by Eliche-Quesada et al. [17], who found that the water absorption of bricks increased with the proportion of marble in raw materials because marble contained a large amount of CaCO_3_. 

**Figure 2:**
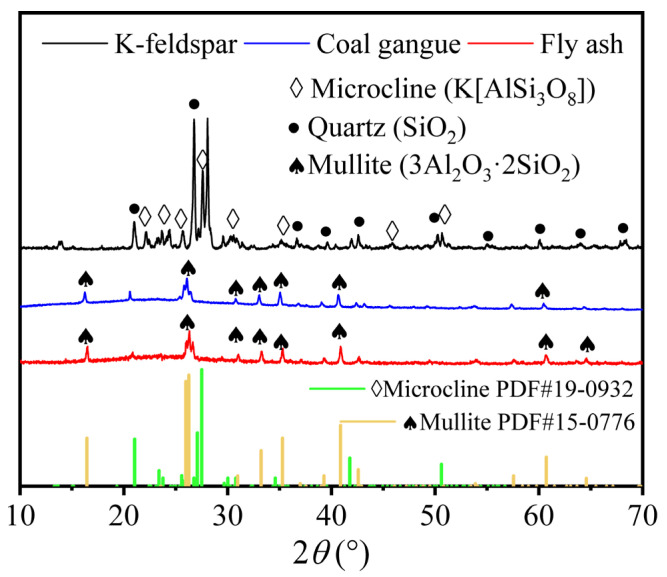
XRD spectra of coal gangue, K-feldspar and fly ash.

**Figure 3:**
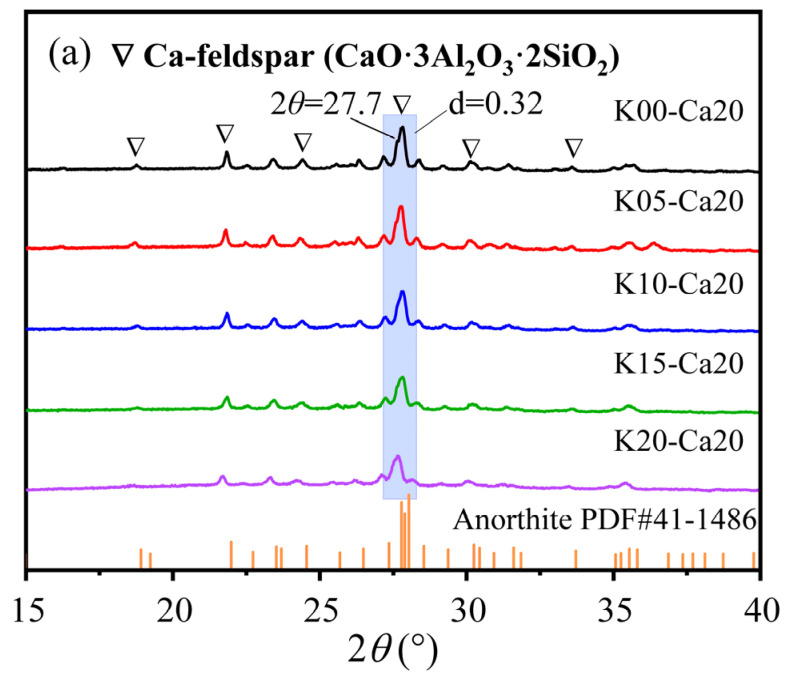

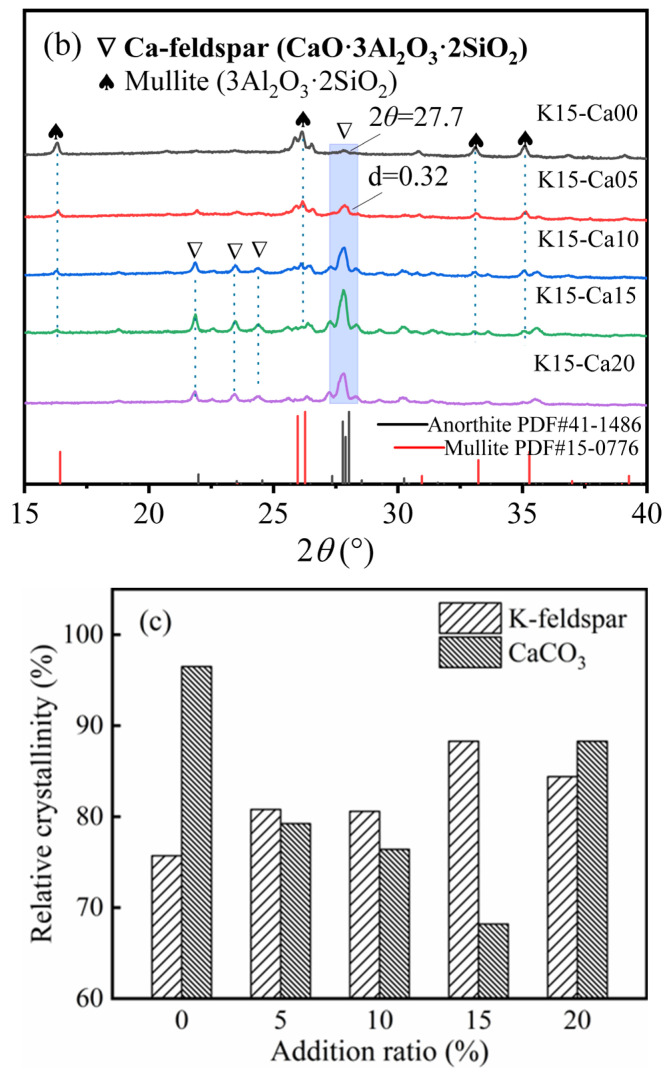
The XRD spectra of insulation bricks produced at different K-feldspar and CaCO_3_ addition ratios (**a**,**b**) and the relative crystallinity of Ca-feldspar (**c**) (purple region represents Ca-feldspar’s peak).

**Figure 4:**
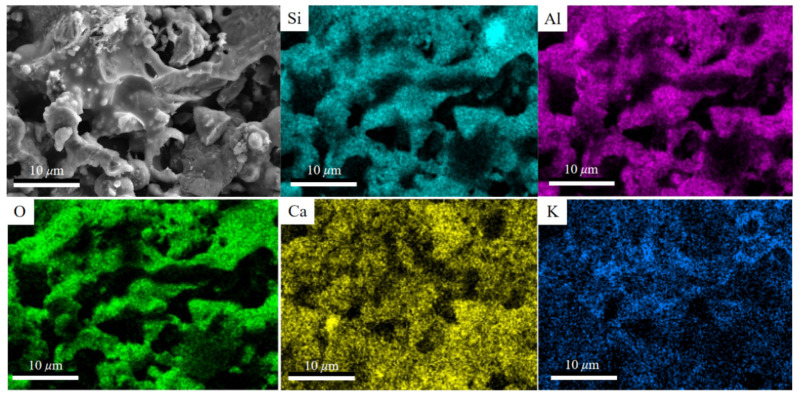
SEM image and EDS mapping of the cross-section of K15-Ca10 treatment.

**Figure 5:**
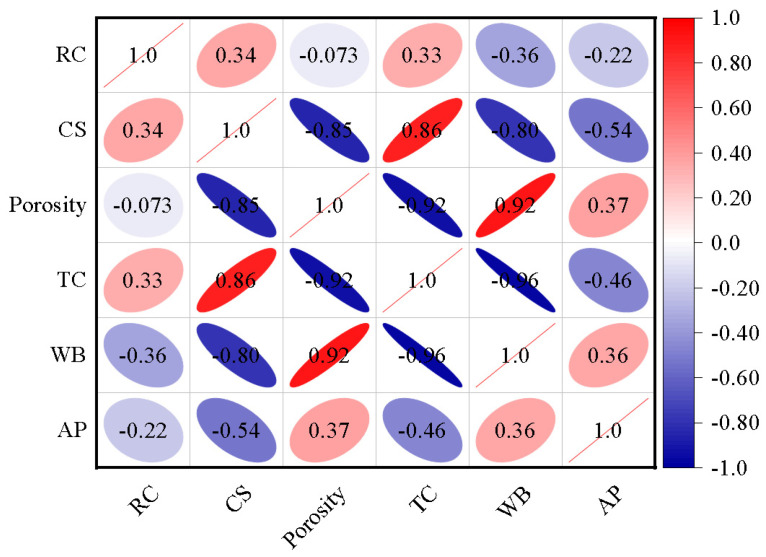
Correlation analysis of different indexes of insulation bricks prepared with different K-feldspar and CaCO_3_ addition ratios (RC: relative crystallinity; CS: compressive strength; TC: thermal conductivity; WB: water absorption; AP: average pore size).

The crystals in K-feldspar contained quartz and microcline, while gangue and fly ash mainly contained mullite (Figure 2). After sintering, a new crystalline Ca-feldspar substance (Figure 3a,b) appeared in the insulation brick, and this was because SiO_2_ and Al_2_O_3_ in gangue, K-feldspar and fly ash melted into the glassy phase under high temperatures, reacting with CaO and producing Ca-feldspar. Ca-feldspar is a kind of plagioclase with a molecular formula of CaAl_2_Si_2_O_8_, and it generally exists in nonmetallic stable phases [26]. The SEM-EDS image of K15-Ca10 treatment (Figure 4) showed that four elements, Si, Al, O and Ca, are located basically in the same position, which further supports the existence of Ca-feldspar. The relative crystallinity of Ca-feldspar crystals (Figure 3c) was calculated via Ca-feldspar peaks (purple region in Figure 3a,b). Relative crystallinity was defined as the ratio of crystalline peaks to non-crystalline peaks. Using MDI Jade 6.5 software, the peaks were fitted and calculated using the ratio of half-height width to relative intensity (RIR). When the ratio of CaCO_3_ was fixed at 20%, the relative crystallinity of Ca-feldspar increased first but then dropped, reaching a maximum of 88.3% when K-feldspar’s proportion was 15 wt%. When the K-feldspar ratio was fixed at 15 wt%, the relative crystallinity of Ca-feldspar decreased first but then increased, reaching a minimum of 68.0% when the CaCO_3_ proportion was 15 wt%. The trend of relative crystallinity (Figure 3c) was consistent with that of compressive strength (Figure 1a), and the correlation coefficient was 0.45 (Figure 5). The relative crystallinity of Ca-feldspar seems to be positively correlated with the compressive strength of thermal insulation bricks. This finding was similar to the study by Zouaoui et al. [27], who found that an increase in Ca-feldspar crystallinity helped improve the mechanical strength of bricks.

**Figure 6:**
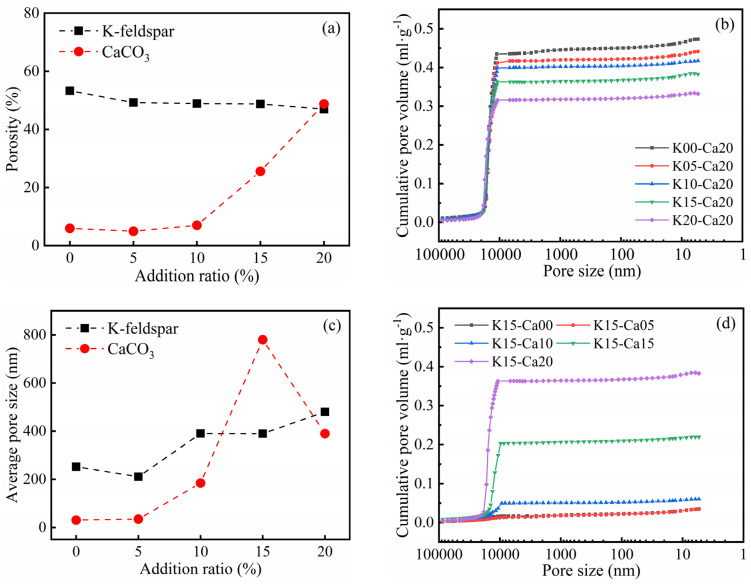
The porosity (**a**), cumulative pore volume (**b**,**d**) and average pore size (**c**) of insulation bricks produced at different K-feldspar and CaCO_3_ addition ratios (K-feldspar: fixed CaCO_3_ addition ratio of 20 wt%; CaCO_3_: fixed K-feldspar addition ratio of 15 wt%).

**Figure 7:**
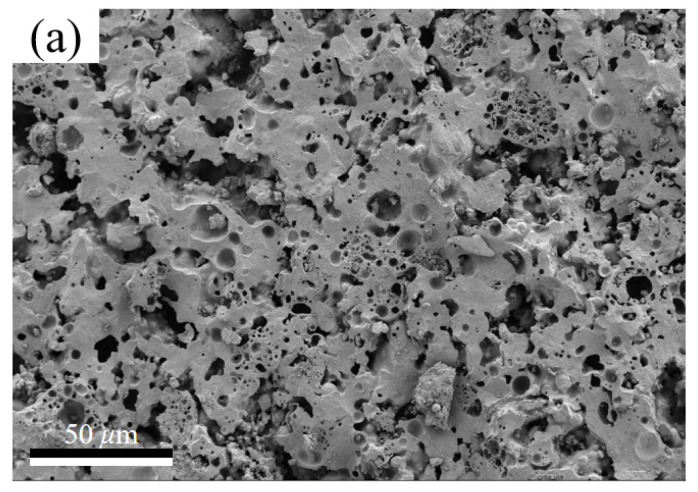

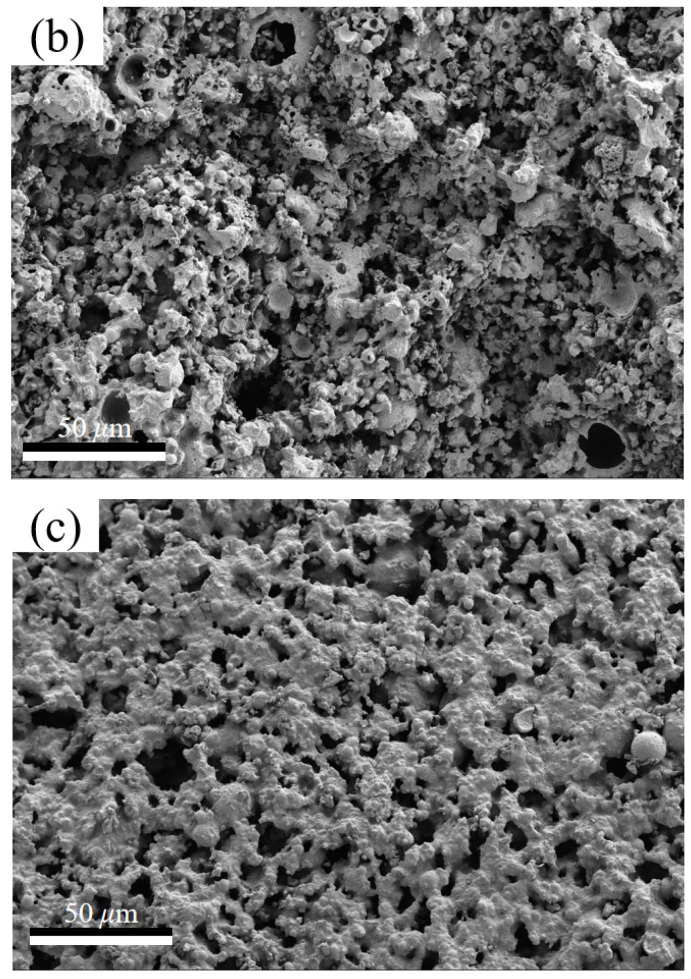
The SEM for the cross-section of K15-Ca00 (**a**), K15-Ca10 (**b**) and K15-Ca20 (**c**) insulation bricks.

The mechanical and thermal insulation properties of bricks were closely related to their internal pore structure. When the ratio of CaCO_3_ was 20 wt%, with an increase in K-feldspar, the porosity of thermal insulation bricks decreased slightly from 53.3% to 47.0% (Figure 6a), the cumulative pore volume decreased from 0.47 mL g^−1^ to 0.33 mL g^−1^ (Figure 6b), and the average pore diameter increased from 252.6 nm to 480.2 nm (Figure 6c). This indicated that the molten flux, like K-feldspar, had a pore-filling effect. This is consistent with the findings of Dai et al. [20], who found that an increase in Na_2_SiO_3_ was beneficial in reducing the porosity of bricks because it filled micropores and mesopores. When the ratio of K-feldspar was fixed at 15%, with an increase in CaCO_3_, the porosity of thermal insulation bricks increased to 48.8% (Figure 6a), the cumulative pore volume increased to 0.38 mL g^−1^ (Figure 6d), and the average pore size reached a maximum value of 780 nm (Figure 6c), which was attributed to the porogenic effect of CO_2_. Barnabas et al. [28] produced energy-efficient bricks by adding 10 wt% walnut shells to clay and found that the thermal decomposition of the organic matter in walnuts produced large amounts of CO_2_ gas, increasing the porosity of bricks by 10%. It should be noted that when the CaCO_3_ ratio increased from 15 wt% to 20 wt%, the average pore size decreased, probably because the decomposition of excess CaCO_3_ produced a large amount of Ca-feldspar that caused a dense distribution of small pores inside the insulation brick. The pores in K15-Ca10 and K15-Ca20 were more intense than K15-Ca00 (Figure 7), and the pores in K15-Ca20 were the most dense and uniformly distributed among the three treatments. Comparing Figure 1, Figure 5 and Figure 6, it could be observed that porosity was negatively correlated with thermal conductivity and positively correlated with the water absorption, and the average pore size was negatively correlated with compressive strength. Wu et al. [29] prepared sintered bricks with sludge and shale, and they found that porosity was correlated with both compressive strength and thermal conductivity. Abjaghou et al. [30] suggested that CaCO_3_ had a pore-forming effect, and thermal conductivity could be predicted from pore data using Landauer and Maxwell–Eucken correlation analyses. 

In summary, when the addition ratios of K-feldspar and CaCO_3_ were selected as 15 wt% and 10 wt%, respectively, the compressive strength of thermal insulation bricks was 22.5 MPa, and thermal conductivity was only 0.39 W m^−1^ K^−1^, which showed the best overall performance. Moreover, the MU15.0 strength grade (>15.0 MPa) and 0.40 heat transfer coefficient grade (0.31~0.40) stipulated in GB/T 26538-2011 [31] were attained. During the sintering process, the CO_2_ derived from CaCO_3_ resulted in the brick’s porous structure and reduced its thermal conductivity; meanwhile, K-feldspar promoted the formation of Ca-feldspar and increased the compressive strength of thermal insulation bricks.

### 3.2. Sintering Temperature Affects the Silicate Polyhedral and Porous Structure of Thermal Insulation Bricks

**Figure 8:**
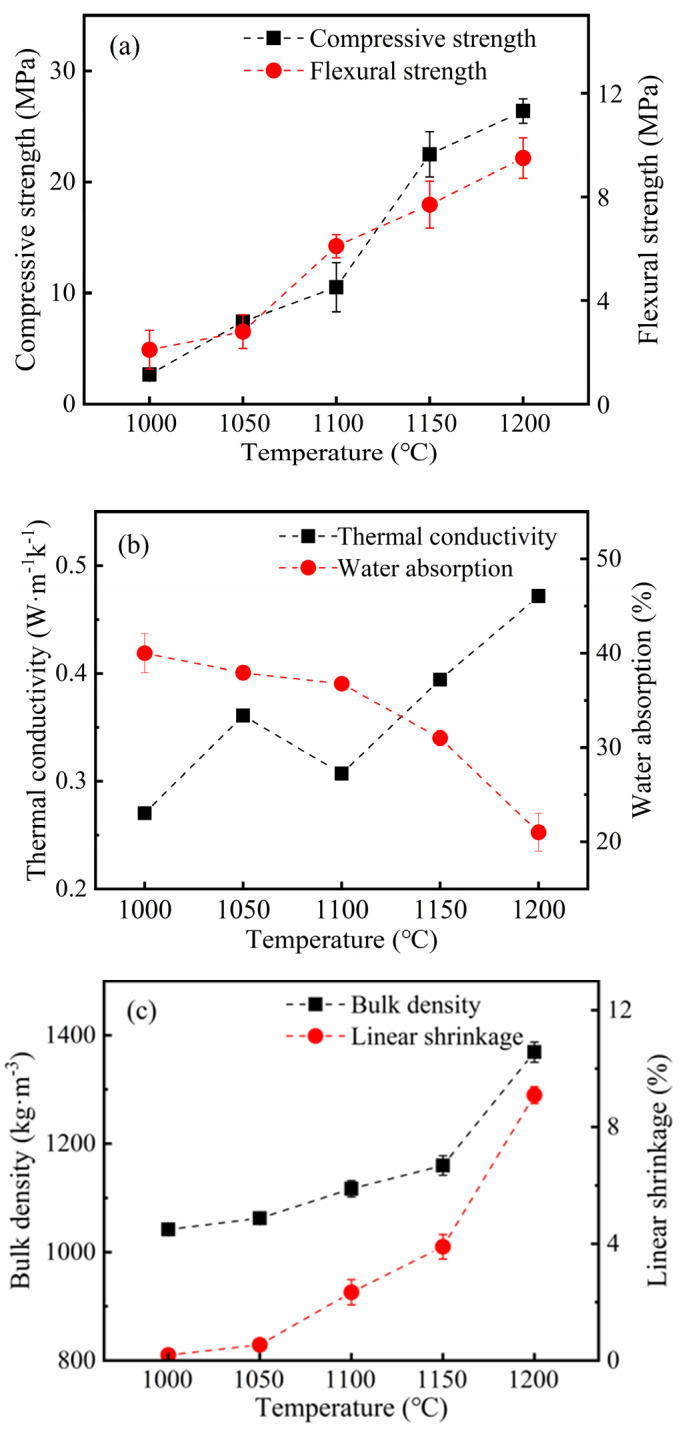
Compressive strength and flexural strength (**a**), thermal conductivity and water absorption (**b**), and bulk density and linear shrinkage (**c**) of insulation bricks produced at different sintering temperatures.

Based on the above results, when the optimal sintering temperature was explored, the ratios of K-feldspar and CaCO_3_ were set at 15 wt% and 10 wt%, respectively. As sintering temperatures increased from 1000 °C to 1200 °C, the compressive strength of thermal insulation bricks increased from 2.67 MPa to 26.4 MPa, flexural strength increased from 2.1 MPa to 9.5 MPa (Figure 8a), the water absorption decreased from 40.0% to 21.1%, and thermal conductivity increased from 0.27 W m^−1^ k^−1^ to 0.47 W m^−1^ k^−1^ (Figure 8b). In addition, bulk density and sintering shrinkage both increased gradually with an increase in sintering temperature, reaching 1369 kg m^−3^ and 9.1% at 1200 °C, respectively (Figure 8c). Luo et al. [32] produced composite sintered bricks using iron waste and coal gangue powder and found that when temperatures increased from 950 °C to 1150 °C, the bulk density of bricks increased from 1.62 g cm^−3^ to 1.65 g cm^−3^. Cheng et al. [15] suggested that as sintering temperatures increased from 950 °C to 1100 °C, the compressive strength of bricks increased to 19.8 MPa. The reason was that at low sintering temperatures, K-feldspar did not completely melt into a glassy phase but existed in an incomplete sintered state, which could not promote the formation of Ca-feldspar. The content of Ca-feldspar was positively correlated with the compressive strength of thermal insulation bricks, and the lack of Ca-feldspar led to low compressive strength. In summary, the high-temperature condition was beneficial in producing thermal insulation bricks with high compressive strength. 

**Figure 9:**
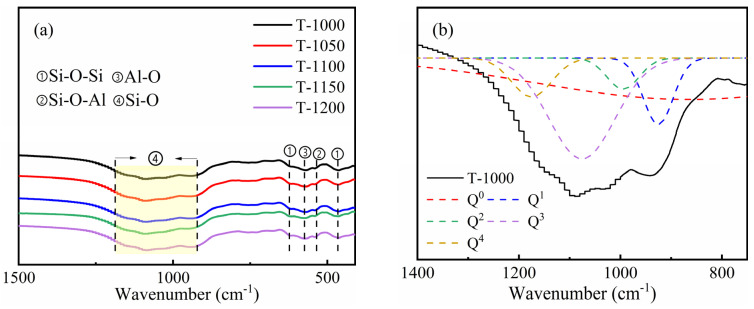

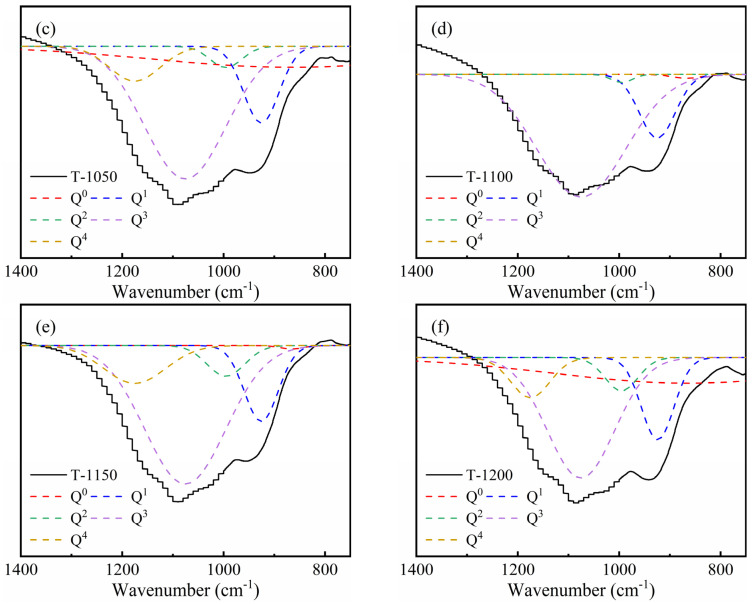
The FTIR spectra of insulation bricks produced at different sintering temperatures (**a**) and the split peaks fitted to Si–O peak (yellow region) (**b**–**f**).

In order to investigate the mechanism of sintering temperatures on the formation of silicate polyhedrons inside thermal insulation bricks, brick samples were analyzed via FTIR and XRD. The FTIR results of insulating bricks produced at different sintering temperatures are shown in Figure 9a. The peaks of 470 cm^−1^ and 620 cm^−1^ show the bending vibration of Si–O–Si [21], 540 cm^−1^ is the bending vibration of Si–O–Al [33], and 800~1300 cm^−1^ (yellow part) is the asymmetric stretching vibration region of the bridging oxygen bond and non-bridging oxygen bond. A bridging oxygen bond means that an O atom is linked with Ca/Mg at one end and Si/Al at the other end. A non-bridging oxygen bond means that an O atom is linked with Si/Al at both ends. The Si–O structure in glass contained reticulated SiO_2_, lamellar [Si_2_O_5_]^2−^, chain-structured [Si_2_O_6_]^4−^, dimer-like [Si_2_O_7_]^6−^ and monomeric [SiO_4_]^4−^ [34]. The polymerization degree of the Si–O network structure was evaluated via Q_n_ (n represented the amount of bridging oxygen of [SiO_4_]) [35]. Q_0_, Q_1_, Q_2_ and Q_3_ represented the structural units of SiO_4_^4−^, Si_2_O_7_^6−^, Si_2_O_6_^4−^ and Si_2_O_5_^2−^, respectively, and their corresponding wave numbers were 840~890 cm^−1^, 900~950 cm^−1^, 960~1030 cm^−1^, 1050~1100 cm^−1^ and 1160~1190 cm^−1^, respectively [36]. The yellow part of the curves was fitted using the peakfit program (Figure 9b–f) [21], and the polymerization degree of the Si–O network structure was calculated via the formula NBO/T = 4 × Q_0_ + 3 × Q_1_ + 2 × Q_2_ + 1 × Q_3_ (Table 3). NBO/T indicates the polymerization degree of the glass network structure in the brick. Higher ratios denote lower polymerization degrees, while lower ratios denote higher polymerization degrees. NBO/T values changed from 2.69 to 1.24 when sintering temperatures increased from 1000 °C to 1150 °C, which meant that the polymerization degree of the Si–O network’s structure increased. However, when the temperature increased to 1200 °C, NBO/T increased to 2.23, which indicated that 1150 °C was the most optimum sintering temperature to obtain a high degree of Si–O network structure polymerization. 

**Figure 10:**
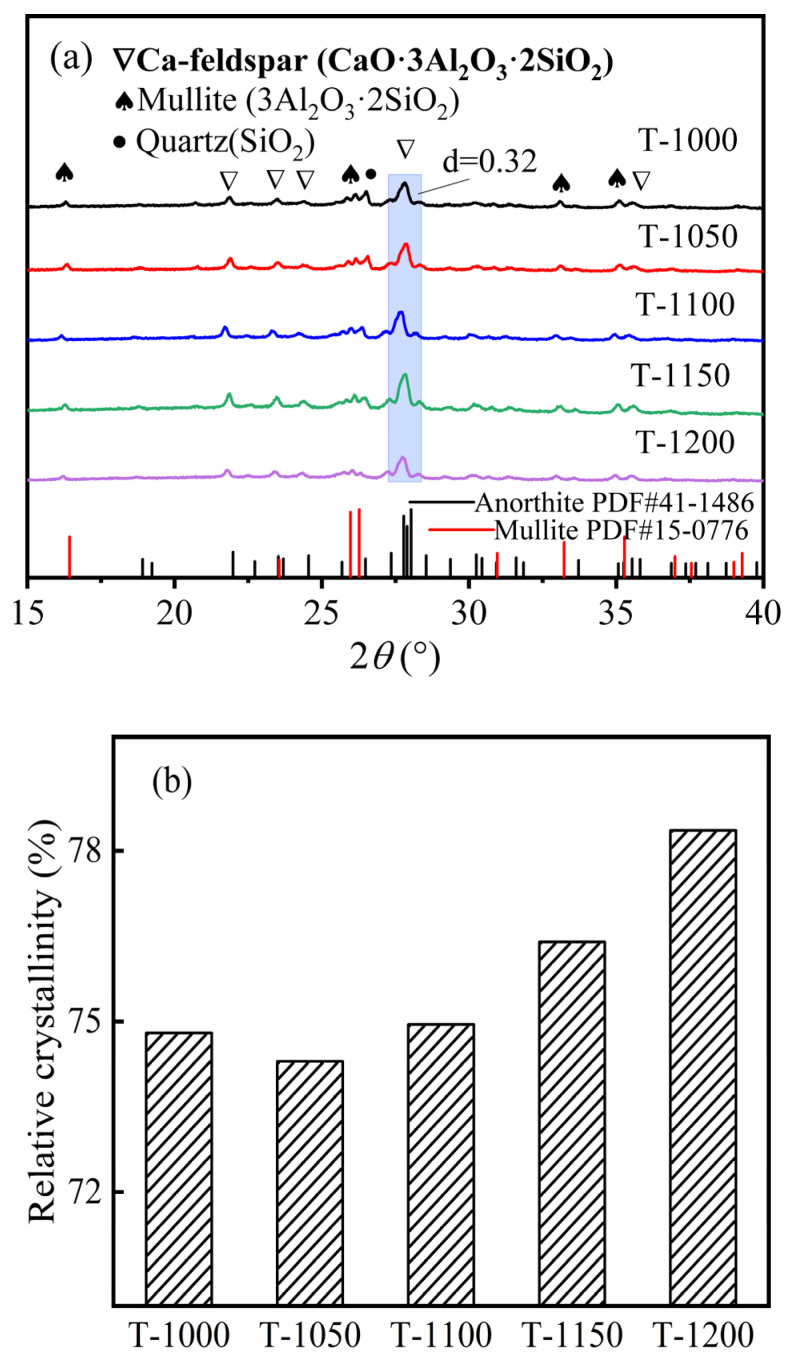
The XRD spectra of insulation bricks produced at different sintering temperatures (**a**) and the relative crystallinity of Ca-feldspar (**b**).

XRD results showed that with an increase in sintering temperatures, the relative crystallinity of Ca-feldspar increased and reached a maximum of 78.4% at 1200 °C (Figure 10b), indicating that higher sintering temperatures were favorable for the formation of Ca-feldspar. Although the relative crystallinity of Ca-feldspar decreased slightly when temperature increased from 1000 °C to 1050 °C (Figure 10b), the compressive and flexural strengths of thermal insulation bricks still increased (Figure 8a); the possible reason for this is that the contribution of the Si–O network’s polymerization degree to mechanical strength was greater than that of Ca-feldspar. When the sintering temperature was higher than 1150 °C, due to the formation of large amounts of Ca-feldspar, its contribution to the mechanical strength gradually exceeded that of the Si–O network’s polymerization degree.

**Figure 11:**
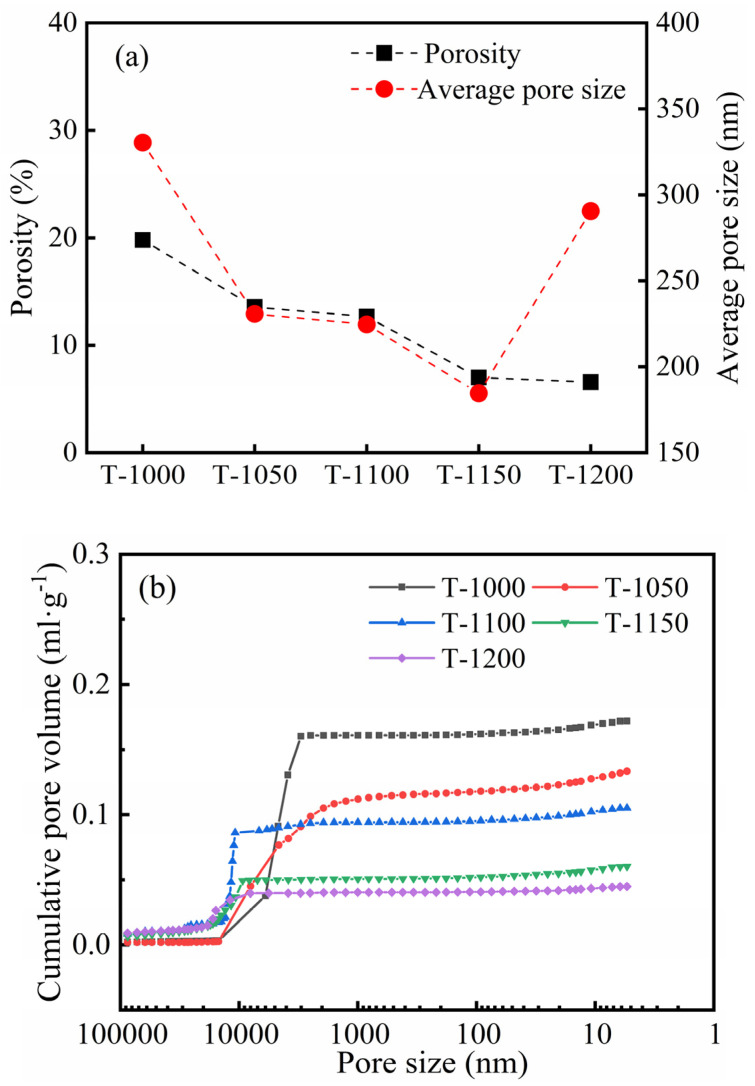
The porosity, average pore size (**a**) and cumulative pore volume (**b**) of insulation bricks produced at different sintering temperatures.

**Figure 12:**
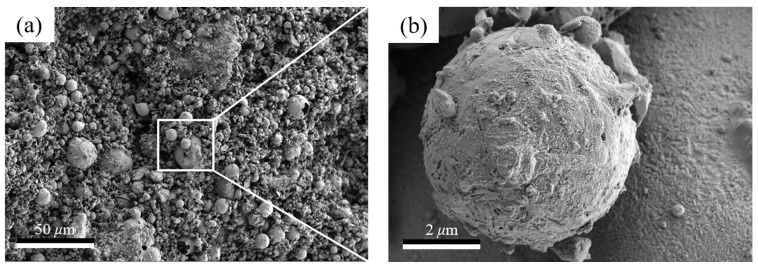

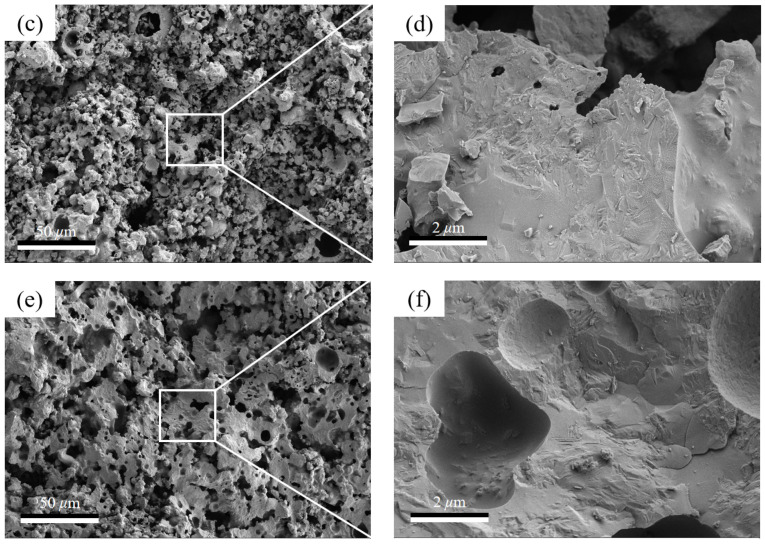
SEM images of the cross-section of insulating bricks produced at 1000 °C (**a**,**b**), 1150 °C (**c**,**d**) and 1200 °C (**e**,**f**).

As sintering temperatures increase from 1000 °C to 1200 °C, the porosity of thermal insulation bricks gradually decreased from 19.8% to 6.6% (Figure 11a), and the cumulative pore volume decreased from 0.17 mL g^−1^ to 0.04 mL g^−1^ (Figure 11b). High sintering temperatures promoted an increase in capillary pressure and surface tension of the glassy phase, destroying part of the pore structure, which ultimately led to a decrease in porosity and a denser brick [6]. The decrease in porosity favored heat conduction, which was the reason that the thermal conductivity of thermal insulation bricks was positively correlated with sintering temperatures [37,38]. With an increase in sintering temperatures to 1150 °C, the average pore size decreased from 330.4 nm to 184.6 nm (Figure 11a) due to the transition of the brick billet from an incompletely sintered state to an optimally sintered state, where mineral powder fused into a glassy phase, reducing the average pore. However, when sintering temperatures increased to 1200 °C, the average pore size increased to 290.6 nm (Figure 11a) because small pores merged into large pores after fully molten glassy phases stuck to each other. The internal morphology of thermal insulation bricks produced at 1000 °C, 1150 °C and 1200 °C is shown in Figure 12. Thermal insulation bricks produced at 1000 °C contained a large number of spherical mineral particles (Figure 12a,b), which comprised an incompletely sintered state of mineral powder. As for 1150 °C (Figure 12c,d), the mineral powder was sufficiently fused and bonded together to form a skeleton-like porous structure. At 1200 °C, a large number of small pores merged into large pores, and the densification of thermal insulation bricks was the highest (Figure 12e,f).

## 4. Conclusions

This study proposed an innovative method for the resource utilization of coal gangue, i.e., the production of high-strength thermal insulation bricks with the assistance of K-feldspar, CaCO_3_ and fly ash. The results showed that the optimum addition ratios of coal gangue, K-feldspar, CaCO_3_ and fly ash were 65%, 15%, 10% and 10%, respectively, and the optimum sintering temperature was 1150 °C. Thermal insulation bricks produced under this condition had a compressive strength of 22.5 MPa and thermal conductivity of 0.39 W m^−1^ k^−1^. During the sintering process, CaCO_3_ was decomposed into CO_2_ and CaO; then, CO_2_ promoted the formation of a porous structure and reduced the thermal conductivity. Meanwhile, K-feldspar facilitated the conversion of CaO into Ca-feldspar, increasing its mechanical strength. 

In previous studies, gangue has generally been backfilled into the ground or ground into a powder and mixed with cement as an active agent. However, the former method still has the risk of contaminating soil and groundwater, and the latter method cannot utilize a large amount of gangue because the mixing ratio of gangue in cement is too low. In summary, the use of coal gangue to produce high-strength insulation bricks not only solved the problem of coal gangue’s occupation of land and pollution of the environment but also produced excellent construction materials with high practical application value.

## Figures and Tables

**Table 1:** Ratio of various raw materials for thermal insulation bricks.

	K-Feldspar (%)	Fly Ash (%)	Coal Gangue (%)	CaCO_3_ (%)
K00-Ca20	0	10	70	20
K05-Ca20	5	10	65	20
K10-Ca20	10	10	60	20
K15-Ca20	15	10	55	20
K20-Ca20	20	10	50	20
K15-Ca00	15	10	75	0
K15-Ca05	15	10	70	5
K15-Ca10	15	10	65	10
K15-Ca15	15	10	60	15
K15-Ca20	15	10	55	20

**Table 2:** Main ingredients of raw materials (wt%).

	Coal Gangue	K-Feldspar	Fly Ash
SiO_2_	39.7	64.1	45.4
Al_2_O_3_	43.0	17.6	33.5
Fe_2_O_3_	6.16	2.64	8.80
CaO	4.06	2.23	5.75
K_2_O	1.78	8.86	1.84
ZnO	0.04	/	0.05
MgO	0.49	0.37	0.63
Na_2_O	0.31	3.59	0.30
TiO_2_	2.70	0.16	/
MnO	0.08	0.06	0.07
P_2_O_5_	0.30	0.03	0.39
SO_3_	1.08	0.03	1.07

**Table 3:** Calculation results of NBO/T.

Sample	Q^0^	Q^1^	Q^2^	Q^3^	NBO/T
T-1000	51.4	8.61	4.36	28.9	2.69
T-1050	26.1	12.5	3.16	50.0	1.98
T-1100	0.82	16.8	1.25	81.1	1.37
T-1150	0.46	14.8	6.71	64.6	1.24
T-1200	33.1	13.3	5.47	40.4	2.23

## Data Availability

Data are contained within the article.
